# Retrohepatic Mass: A Case of Human Herpesvirus-8 Negative Multicentric Castleman’s Disease

**DOI:** 10.7759/cureus.16848

**Published:** 2021-08-03

**Authors:** Hefei Liu, Danyon J Anderson, Harpreet Gill, Pinky Jha

**Affiliations:** 1 Medicine, Medical College of Wisconsin, Wauwatosa, USA; 2 Hospital Medicine, Medical College of Wisconsin, Wauwatosa, USA; 3 Internal Medicine, Medical College of Wisconsin, Wauwatosa, USA

**Keywords:** multicentric castleman’s disease, mcd, anemia of chronic diseases, polycystic ovarian syndrome, lymphoproliferative disorder

## Abstract

Multicentric Castleman's disease (MCD) is a rare lymphoproliferative disorder with aggressive systemic presentation and poor prognosis. Here, we present a case of MCD in a 37-year-old Asian American woman with a past medical history of the polycystic ovarian syndrome (PCOS), human papilloma virus (HPV), herpes simplex virus-1 (HSV-1), iron deficiency, and vitamin B_12_ deficiency-related anemia. The patient underwent surgical resection with good recovery. Hemoglobin and erythrocyte sedimentation rate (ESR) normalized after surgical resection. Although the influence of risk factors such as human immunodeficiency virus (HIV) or human herpesvirus-8 (HHV-8) infections on MCD relapse are not well understood, patient education on MCD risk factors is important, as they may place the patient at greater risk for recurrence. MCD should be considered in patients with chronic inflammation and a mass on imaging.

## Introduction

Castleman’s disease (CD) is a group of rare lymphoproliferative disorders due to hypersecretion of IL-6, leading to cytokine dysregulation, systemic lymphadenopathy, and inflammation [1-2]. CD is a relatively rare disease with 404 published cases. Most CD patients are women (60%). The median age of diagnosis is 34 years with a range from 2 to 74 years. Of the published CD cases, 68% were unicentric Castleman’s disease (UCD); UCD has an incidence of 15 per million patient-years [3]. Multicentric Castleman's disease (MCD), a subtype of CD, has an aggressive systemic presentation with a poor prognosis due to complications including infection, lymphoma, paraneoplastic pemphigus, plasma cell dyscrasias, and POEMS (polyneuropathy, organomegaly, endocrinopathy, M protein, and skin changes) [1, 4]. Human herpesvirus-8 (HHV-8) is strongly associated with MCD and a positive HHV-8 serology increased the index of suspicion for this otherwise rare disease. However, MCD may rarely be idiopathic with an incidence of five per million patient-years [3]. Pharmacotherapy via corticosteroids and targeted immunotherapy of IL-6 and CD-20 is efficacious, but surgery is still indicated for symptom control [5-6]. Here we present a case of idiopathic, HHV-8 negative MCD with a complicated retrohepatic tumor.

## Case presentation

A 37-year-old Asian American woman with a past medical history significant for the polycystic ovarian syndrome (PCOS), human papilloma virus (HPV), herpes simplex virus-1 (HSV-1), iron deficiency, and vitamin B12 deficiency-related anemia presented in February to the emergency department (ED) with body aches, fever, fatigue, cough, and recurrent upper respiratory infections for the past three months.

**Figure 1:**
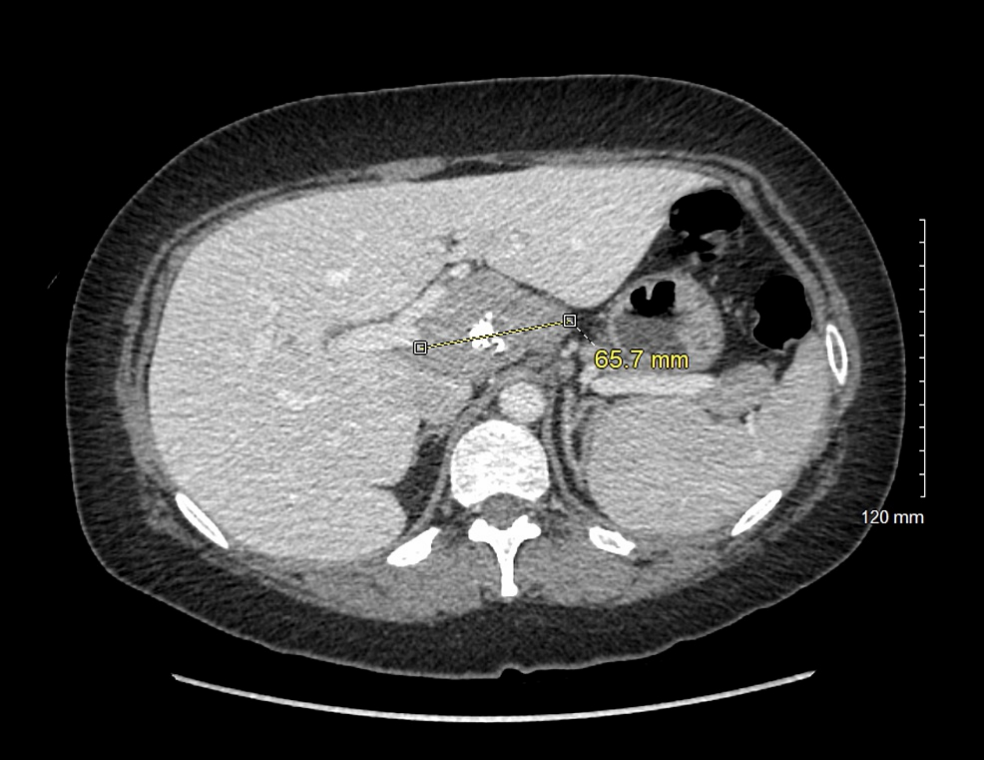
A partially calcified soft tissue mass of uncertain etiology with several adjacent prominent upper abdominal lymph nodes. Differential included a conglomerate nodal mass such as primary hematologic malignancy or metastatic nodal adenopathy, primary sarcoma, and an exophytic mass arising from the pancreas or the hepatic caudate lobe.

Two weeks later, the patient presented to the hematology clinic for a general follow-up regarding her long-term iron malabsorption/deficiency, vitamin B12 deficiency-related anemia, and increasing joint pain in her elbows, knees, and wrists which worsened over the course of the day. Coronavirus disease 2019 (COVID-19) was suspected but the serology test returned negative. Elevations in C-reactive protein (CRP), erythrocyte sedimentation rate (ESR), and +antinuclear antibody (ANA) warranted a CT scan of the chest, abdomen, and pelvis in April. The scan showed an incidental finding of a 65.7 mm porta hepatis mass on the posterior of the left portal vein, posterior to the right and common hepatic arteries, and the associated retroportal lymph nodes (Figure 1). Her lactate dehydrogenase (LDH), vitamin B12, and white blood cell (WBC) counts were within limits. She denied any fever, alcohol use, or illicit drug use. More specific tests including hepatitis B, hepatitis C, parietal cell antibodies, and intrinsic factor antibodies were negative. To rule out neoplastic etiologies, the carcinoembryonic antigen (CEA) and cancer antigen (CA) 19-9 were measured and were found to be within normal ranges. Infectious etiologies were also considered but ultimately excluded due to negative blood and urine labs. Biopsy results of the hepatic mass were inconclusive but suggested several differential diagnoses of a lymphoproliferative disorder, a ganglioneuroma, or a schwannoma such as lymphoid or autonomic neural origin.

Surgery was performed in August without complications. The hepatic artery lymph node, the lymph node at the right crus diaphragm, and the retroportal lymph node and tumor were excised, all of which were HHV-8 negative. Microscopy studies showed lymph nodes with varying degrees of florid follicular hyperplasia and progressive transformation of germinal centers. Rare atretic follicles, as well as follicles with multiple germinal centers, were also observed. The interfollicular areas were expanded by increased plasma cells without atypia as well as several foci of vascular proliferation. While no vesicular hyalinization was observed, these findings were used to diagnose MCD.

Post diagnosis, the patient was managed with symptomatic observation along with continued monitoring of ESR and immunoglobulin G (IgG). Repeat abdominal CT was done every six months to track remission and to determine candidacy for siltuximab therapy in the event of symptom exacerbation. Hemoglobin and ESR normalized after surgical resection. To date, the patient continues to recover well after her surgery and has plans to resume activities of daily living.

## Discussion

Castleman’s disease presents with a wide range of clinical presentations and associated risk factors. Clinical presentation often includes enlarged lymph nodes, fever, night sweats, loss of appetite, and weight loss. Blood tests generally indicate elevated levels of IL-6 while also ruling out other potential etiologies such as infection and autoimmune conditions. Affected lymph nodes may be identified with imaging tests and subsequently biopsied to exclude lymphoma and other lymphadenopathies [7].

There are multiple underlying etiologies of MCD. HHV-8 associated MCD is often seen in immunocompromised individuals and is believed to be driven by HHV-8 driven cytokine release within lymph nodes, resulting in clinical symptoms. POEMS-associated MCD is observed in patients with polyradiculoneuropathy, organomegaly, endocrinopathy, monoclonal plasma cell disorders, and skin changes, and is likely due to cytokine production from dysregulated monoclonal plasma cells. The causes of idiopathic MCD may involve multiple dysregulated immune processes leading to cytokine overproduction. Other potential causes of idiopathic MCD have been discussed and are possibly due to autoimmunity, neoplasms, and viral infection [8].

The MCD therapy varies depending on the etiology and severity of symptoms [9]. Highly active antiretroviral therapy is often indicated for HIV treatment in HHV-8 associated MCD. Idiopathic MCD has been usually managed acutely with steroids to prevent symptomatic flares. Immunomodulatory agents such as tocilizumab, cyclosporin, rituximab, and other cytotoxic agents may be prescribed for chronic therapy.

Here we present a rare, idiopathic case of MCD with potential associations with anemia of chronic disease and PCOS. Chronically elevated levels of IL-6 in MCD and PCOS may be linked to increased hepcidin expression in the liver, leading to anemia of chronic disease [10-13]. This may explain the normalization of our patient’s hemoglobin and ESR following surgical resection of the mass.

The patient's management will be continued as described above, and IL-6 inhibitors such as siltuximab will be considered if the patient’s symptoms worsen [2, 14]. Siltuximab has been shown to be well tolerated for long-term therapy for MCD [15]. Additionally, siltuximab added to a supportive care regime has yielded better results in alleviating symptoms than a supportive care regime alone. Surgical resection may also be revisited in the future.

Although the influence of risk factors such as HIV or HHV-8 infections on MCD relapse is not well understood, patient education on MCD risk factors is important, as they may place the patient at greater risk for disease recurrence.

This case of MCD describes a mass uniquely located in the retrohepatic region, which complicated the surgical excision. Additionally, elevated levels of IL-6 potentially caused by both PCOS and MCD suggest an interesting concurrency in affecting the presentation of the patient’s general symptoms.

## Conclusions

This case of MCD is unique due to its HHV-8 negativity, the unusual location of the tumor, as well as its associations with other chronic conditions related to elevated levels of IL-6. Chronically elevated levels of IL-6 in both MCD and PCOS may be linked to increased hepcidin expression in the liver, leading to anemia of chronic disease, which may explain the normalization of our patient’s hemoglobin and ESR following surgical resection of the mass. MCD should be considered in patients with chronic inflammation and a mass on imaging.
